# Eddy Current Measurement of Electrical Resistivity in Heat-Treated Zr-2.5%Nb Pressure Tubes

**DOI:** 10.3390/s24237426

**Published:** 2024-11-21

**Authors:** W. G. Thorpe, P. R. Underhill, T. W. Krause

**Affiliations:** 1 Department of Physics, Engineering Physics and Astronomy, Queen’s University Kingston, Kingston, ON K7L 3N6, Canada; 16wgt1@queensu.ca; 2 Department of Physics and Space Science, Royal Military College of Canada, Kingston, ON K7K 7B4, Canada; ross.underhill@rmc.ca

**Keywords:** Zr-2.5%Nb, pressure tubes, eddy current testing, electrical resistivity, fuel channels

## Abstract

Zr-2.5%Nb pressure tubes (PTs) house uranium fuel bundles in the fuel channels of CANDU^®^ nuclear reactors. Preventing a failure mode caused by contact of the PT with an outer calandria tube (CT) is performed by inspection using eddy current (EC) testing and ultrasonic testing (UT) to measure the PT-CT gap. EC gap measurements are particularly sensitive to circumferential variation of the PT’s electrical resistivity due to microstructural variations. A full-factorial experiment was performed to examine the statistical significance of variations in the EC test parameters and manufacturing conditions on the average circumferential electrical resistivity of as-manufactured PTs. It was found that 79% of the variance in the data could be attributed to variations caused by any of the test factors or combinations of test factors. The parameters that accounted for the majority of the variance were: (1) heat treatment (HT); (2) HT and EC frequency; (3) probe inner or outer surface placement; and (4) EC frequency. Measurements of circumferential resistivity showed up to ±2.3% variation from the average of either surface. HT caused the average PT resistivity to decrease at a rate of $1.53\pm 0.08\;\frac{\mu \Omega \cdot cm}{\log\left(hr\right)}$ and $1.1\pm 0.4\frac{\mu \Omega \cdot cm}{\log\left(hr\right)}$ for inner and outer PT surfaces, respectively. The results are correlated with differences reported in the literature in the average $\beta_{Zr}$ ribbon thickness in the axial-transverse cross-section between inner and outer PT surfaces. The results demonstrate potential for EC-based resistivity measurements to characterize variations and changes in the microstructure of Zr-2.5%Nb PT material.

## 1. Introduction

Pressure tubes (PTs) are used to house uranium fuel bundles and pressurized heavy water coolant in the fuel channels of CANadian Deuterium Uranium (CANDU^®^) nuclear reactors [1]. These PTs are composed of Zr2.5%Nb, a zirconium alloy containing 2.4–2.8 percentage by weight of niobium (Nb) [2]. Each fuel channel consists of a PT held within a larger diameter calandria tube (CT) by a series of four garter spring spacers. This CT separates the cooler (~70 °C) heavy water neutron moderator via a gas annulus from the pressurized heavy water heat transport system flowing through the PTs [3]. The operating conditions that PTs are subject to include heavy water coolant temperatures between 250 and 300 °C and pressures between 10.5 and 9.9 MPa that vary from the inlet to the outlet, respectively [3]. As a result of this pressure gradient, there is 65 MPa of axial stress in the PT wall and hoop stresses vary from 122 to 130 MPa from the inlet to the outlet, respectively [3,4]. In addition, PTs are also subject to neutron flux irradiation that varies from the inlet to the outlet, with a peak intensity halfway along the tube length of $3.5\times 10^{17}\;n\cdot m^{2}\cdot s^{-1}$, and with energies greater than 1 MeV [4]. These operating conditions cause PTs to undergo various dimensional changes over time, including axial elongation, diametral expansion, sag, and wall thinning [3].

Standards, published by the Canadian Standards Association (CSA), govern the PT design specifications, manufacturing process, operation, and inspection of fuel channels to mitigate the risks of heavy water coolant leaks forming in the PT during operation [5,6,7,8,9,10,11]. One requirement, outlined in [6], is to periodically inspect the PT-CT gap to ensure that the various PT deformation mechanisms do not result in the warmer PT coming into contact with the cooler CT. Due to the horizontal arrangement of the fuel channels, the location of the PT that is most at risk of contact with the CT is the bottom 6 o’clock position. PT-CT contact results in a temperature gradient in the PT, which accelerates deuterium diffusion into the PT at the heavy water coolant interface [12]. Absorption of deuterium into the PT results in hydrogen embrittlement, and a loss of local ductility of the PT [13]. If the solubility limit of deuterium in the PT is exceeded, then hydride blisters begin forming and growing until a critical size, which depends on the local tensile stress, is reached for crack initiation [4]. This crack formation process is referred to as delayed hydride cracking (DHC), and if left unchecked, can lead to coolant leaks in a failure condition known as a loss of coolant accident (LOCA), requiring a costly fuel channel replacement [6,14]. As such, it is paramount to ensure that the PT-CT gap is maintained to prevent this failure mechanism from occurring. Assurance is provided by performing inspections, either by direct measurements of the gap [15], or by the combination of the fuel channel sag measurements and garter spring detection [6].

Non-destructive testing (NDT) techniques that are utilized for monitoring the PT-CT gap include a combination of multi-frequency eddy current (EC) testing (ECT) using a tool called a gap probe and ultrasound [15]. ECT is used for extracting PT-CT gap data [15], garter spring spacer location data for loaded spacers [12,16], and (LISS) nozzle proximity [17,18], while ultrasonic testing (UT) is primarily used for extracting PT wall thickness (WT) data, a crucial parameter for ECT measurements [14]. In addition, ECT also has applications for the inspection of cracks in nuclear steam generator tubes, with Zhao et al. [19] presenting the development of a new hybrid-spiral bobbin EC probe for the detection of arbitrarily oriented cracks.

For measurements of the PT-CT gap, inspection personnel are interested in being able to relate the gap probe’s voltage response to specific fuel channel parameters along a helical scan path with a high degree of precision and accuracy. To this end, there are currently two proposed approaches, with each having its own advantages and disadvantages. These approaches consist of a proposed deterministic inverse or error minimization algorithm (EMA) by Contant et al. [20], which is based on an analytical model by Klein et al. [14], and a machine learning model using a deep neural network trained on experimental data as presented by Purdy et al. [21] and Darling et al. [22]. The main advantage of using deterministic-based models over machine learning methods is that their accuracy is only dependent on the accuracy of the input parameters. However, a major limiting factor to the accuracy of current PT-CT gap predictions that assume a constant resistivity [15] are uncertainties in the effect of fuel channel operating conditions on PT electrical resistivity. In a sensitivity analysis conducted by Klein et al. [14], using possible ranges of input parameter variations, variations in probe lift-off (LO), PT WT, and PT resistivity were found to be the three largest sources of PT-CT gap uncertainty with PT resistivity variations producing about 0.1–0.2 mm of root mean square (RMS) gap prediction error per μΩ·cm of variation, depending on an EC frequency between 4 and 16 kHz. Therefore, an improved understanding of the effects of fuel channel operating conditions on axial and circumferential PT resistivity variations along the helical travel path of the gap probe is needed to compensate for this uncertainty, like the compensation performed by UT for PT WT variations [6,7]. However, there is currently a lack of published literature that examines or models the effects of fuel channel operating conditions on Zr2.5%Nb electrical resistivity. A good starting point for improving this understanding is characterizing, through a statistical study, the degree to which electrical resistivity can vary in the circumferential direction of as-manufactured PTs. This is a starting point for the next step, which would be to simulate the effects of circumferential variations in temperatures of up 20 °C between the top and bottom of the PT as reported by Rodgers et al. [4] and that of irradiation-induced damage effects on resistivity.

The results of Thorpe et al. [23], showed that there are indeed measurable radial variations in resistivity that appear to become more apparent at higher EC frequencies. Therefore, a natural follow-up question is whether variations in EC test parameters, like the inner or outer surface placement of the coil and inspection frequency, have any statistically significant effects on PT resistivity beyond just inherent variations created by variability in the conditions of the manufacturing process of PTs, which has been shown to lead to microstructural variations [3,24,25,26]. This question is relevant for the measurement of electrical resistivity using ECT, as it will indicate whether separate resistivities should be reported from either the PT surface or EC frequency. PT-CT gap inspections also rely on variations in resistivity in the circumferential direction being minimal, as there is no mechanism in the current gap algorithm to compensate for such variations [15]. The accuracy of the gap values returned by this algorithm relies on empirical models obtained from calibrating the gap probe’s response to PT WT and PT-CT gap variations with resistivity held constant [15]. Therefore, a statistical study seeking to characterize circumferential resistivity variations in as-manufactured PTs will be useful for personnel interested in estimating the amount of gap error that can be expected from this empirical model due to resistivity variations in PTs.

One novel objective of this paper is to characterize the electrical resistivity in the circumferential and radial directions of various independently manufactured PT samples using ECT to determine the testing parameters that have the most statistically significant effect on it. These testing parameters include EC frequency and the surface location of the probe (i.e., whether it is over the outer or inner PT surface). The advantages of using ECT to characterize PT electrical resistivity over other methods are its ability to obtain high-resolution data with minimal required sample preparation and low sensitivity to lift-off variations caused by variations in the zirconium oxide layer due to heat treatment (HT) as reported by Price [27]. An additional novel objective is to determine, through experimental measurements, whether HT at 400 °C has a statistically significant impact on electrical resistivity variation in the circumferential direction or its measurement with the ECT parameters. HT has been shown by Bennett et al. [28] to have an effect on resistivity in the axial direction, with reductions of up to 10% in the as-manufactured nominal PT resistivity of 54 μΩ·cm prior to HT. Ref. [28] also provides images that were obtained using scanning electron microscopy of the Zr2.5%Nb microstructure before and after various stages of HT at 400 °C. The effect on resistivity with time of HT has not been investigated for the circumferential direction. However, Price [29] has reported that HT at 400 °C results in a reduction in the difference between axial and circumferential resistivities over time.

As mandated by CSA N285.4-09 [11], cold-worked PTs can require up to 72 h of autoclave HT to accumulate a sufficient oxide layer thickness to inhibit heavy water-induced corrosion during service. Therefore, this paper also characterizes and tracks these resistivity variations at various stages of applied HT, including this 72-h time period. These results can then be used to determine an upper bound on the amount of error that can be expected to be introduced in PT-CT gap prediction algorithms from variations in the resistivity of newly installed PTs.

Based on the results obtained by Bennett et al. [28], it is expected that HT at 400 °C should result in a resistivity change of $-0.963\frac{\mu \Omega \cdot cm}{\log\left(hr\right)}$ of applied HT time. As demonstrated in their paper [28], this effect correlates strongly with the modeled changes in resistivity due to the decomposition of the $\beta_{Zr}$ phase into $\alpha_{Zr}$ and $\beta_{Nb}$. Holt [3] also reports that the extrusion process results in PTs exhibiting variations in circumferential texture with texture fractions that vary between 0.642 and 0.605 from the front to back ends of the PT, respectively. These textures describe grain orientations, and different lattice planes can have different atomic densities, thereby leading to increased electron scattering and resistivity in these directions [30]. As a result, it is expected that there should be a statistically significant difference in resistivity between PT samples taken from the front and back ends of an extruded PT.

The novel results of this paper include high-resolution measurements of electrical resistivity variation of PTs in the circumferential direction after various stages of applied HT at 400 °C as well as a comparison of these results between different EC frequencies. This paper also explores the statistical significance of variations in EC test parameters like frequency and probe surface placement on the average circumferential electrical resistivity of PTs using a full-factorial experiment. These results are used to estimate the amount of PT-CT gap error that can be expected by not compensating for circumferential resistivity variations in PTs in the current gap algorithm [15].

## 2. Model of Changes in Resistivity Due to Heat Treatment Using the Avrami Equation

In addition, the relationship between changes in electrical resistivity caused by the HT process and fraction complete of $\beta_{Zr}$ decomposition in Zr2.5%Nb is modeled using a similar methodology as outlined by Bennett et al. [28]. The intent of this section is to provide a more rigorous explanation for the determination of the Avrami coefficients reported by Bennett et al. [28]. These results are later used to relate resistivity changes to variations in phase composition in the microstructure.

The relationship between phase transformation and HT time is given by the Avrami equation shown below [31].

(1) $$Y(t)=1-exp(-K\cdot t^{n}),$$

where, Y(t) is the volume fraction of a particular phase as a function of time, and t, K, and n are fitting constants that depend on the properties of the microstructural phases of a material.

A simple method for determining the fitting constants in Equation (1) is to linearize it by isolating the exponential terms and taking the double natural log of both sides of the equation. Linearizing Equation (1) allows these fitting constants to be determined using linear regression, which is simpler to perform than non-linear curve fitting, where the quality of the fit is dictated by the accuracy of a guess at the optimal values of the parameters [32]. The resultant equation of this linearization process is given by:

(2) $$\ln\left(ln\left(\frac{1}{1-Y}\right)\;\right)\;=\ln\left(K\right)+n\cdot ln(t).$$

From this equation, the slope of the line of best fit to the linearized data is n, and the y-intercept is $\ln\left(K\right)$.

A necessary assumption for determining these fitting constants is that there is a linear correlation between wt. % Nb in the $\beta_{Zr}$ phase and its volumetric phase fraction, $f_{\beta }=Y$, during decomposition. This assumption is necessary because the literature on the decomposition process of the $\beta_{Zr}$ phase in Zr2.5%Nb only gives the wt. % Nb concentrations in the $\beta_{Zr}$ phase after various amounts of applied HT time. These results are given by Griffiths et al. [26] in their temperature-time-transformation (TTT) diagram, which shows the relationship between wt. % Nb concentration in the $\beta_{Zr}$ phase as a function of HT time in log hours at various temperatures. This assumption also follows from the derivation and extraction of Avrami fit parameters by Bennet et al. [28].

**Figure 1:**
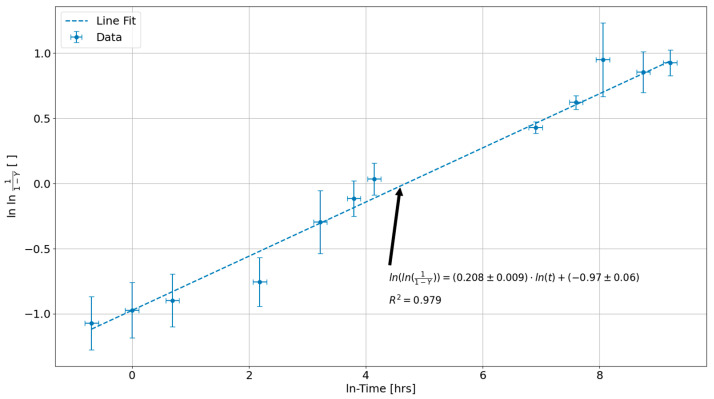
Plot of the % wt. Nb concentration, Y, in the $\beta_{Zr}$ phase of the Zr2.5%Nb alloy as a function of 400 °C HT time in ln(hours). The dashed line corresponds to the line of best fit that minimizes the residual error with the data points. The parameters and uncertainties given by this weighted regression analysis are shown annotated on the graph along with the correlation coefficient given by $R^{2}$.

Now, from the TTT diagram provided by Griffiths et al. [26], the wt. % Nb concentrations in the $\beta_{Zr}$ phase along the 400 °C line are given as ranges, which means that the actual wt. % Nb concentrations and uncertainties must be estimated using the standard practice of taking the true values as the midpoints of the ranges and the uncertainties as half of each range. Mathematically, this process is given by the following equations:

(3) $${\bar{c}}_{Nb}(t)=\frac{c_{Nb}^{u}\left(t\right)+c_{Nb}^{l}\left(t\right)}{2},$$

(4) $$\delta c_{Nb}(t)=\frac{c_{Nb}^{u}\left(t\right)-c_{Nb}^{l}\left(t\right)}{2},$$

where ${\bar{c}}_{Nb}(t)$ is the midpoint or average % wt. Nb concentration as a function of log time in hours, $\delta c_{Nb}(t)$, is the corresponding uncertainty, and $c_{Nb}^{u}(t)$ and $c_{Nb}^{l}(t)$ are the upper and lower bounds on the % wt. Nb concentrations for a particular log base 10 time value, respectively. These concentration values can then be substituted into Equation (2) for Y as it is assumed that the rate of change of the wt. % Nb concentration in the $\beta_{Zr}$ phase and the $\beta_{Zr}$ phase fraction are equal, which therefore makes them interchangeable here for the purposes of extracting a rate of change in the $\beta_{Zr}$ phase decomposition rate. These results are shown plotted in Figure 1, where the time values were converted from the log base 10 time to the natural log time. The dashed line in this plot shows the results obtained using linear regression analysis to generate a line of best fit to the data points. The wt. % Nb concentrations in Figure 1 range between 29% and 92.5%, between the lowest and highest data points.

From the line of best fit in Figure 1, the optimal values for K and n are K=0.38±0.02 and n=0.208±0.009, with K being determined using Equation (3) with this line of best fit’s y-intercept. The uncertainty in K was estimated using error propagation, while the uncertainty in n corresponds to the uncertainty in the slope from the weighted linear regression.

## 3. Materials and Methods

### 3.1. Full-Factorial Experimental Design

For this experiment, a full-factorial design approach is employed with the test factors being:

1. PT sample number;
2. Number of applied HT periods;
3. EC frequency; and
4. Inner or outer surface probe placement.

In such an experiment, the effects of all the possible combinations of the test factors are examined for the response to some variable that depends on each specific combination of test factors [31]. In this case, the variable of interest is the average electrical resistivity measured by the EC probe around the circumference of a PT at a constant axial position. This analysis does not account for the statistical significance of changes in the circumferential positioning of an EC probe on measured PT electrical resistivity, as the circumferential position test factor could not be included here, due to the circumferential orientations of each PT sample during the extrusion manufacturing process being unknown. As a result, the variance in measurements at each of the circumferential positions on each PT sample could not be expected to follow a normal distribution, which is an assumption of this experiment. The main advantages of this full-factorial experimental design approach are that it can be used to determine the sensitivity between different test factor interactions on a response variable, as well as that it allows conclusions to be drawn that are valid over a range of test conditions [31]. The alternative design approach is the one-factor-at-a-time (OFAT) method, which involves designing experiments to only test a single factor at a time [32]. As a result, OFAT experiments have the advantage of being quicker to perform as there are fewer data that must be collected, but ignoring any test factor interactions limits the validity of conclusions that can be drawn from them.

The null hypothesis is relevant to consider for this experiment as it predicts that the results obtained for a response to a given combination of test factors can be attributed to random chance [33]. As a result, no relationship or interaction exists between the given combination of test factors. The mathematical formulation of this hypothesis is given by [33]:

(5) $$\mu_{1}=\mu_{2}=\mu_{3}=\cdots =\mu_{m},$$

where, $\mu_{j}$ is the true mean value of the response variable for test factor combination j, with m defining the total number of combinations of the test factors. It is also assumed that the variances $\sigma_{m}^{2}$ for all test factor combinations must be the same [33]. Therefore,

(6) $$\sigma_{1}^{2}=\sigma_{2}^{2}=\sigma_{3}^{2}=\cdots =\sigma_{m}^{2}=\sigma^{2}.$$

This null hypothesis can only be rejected if the response to some variable for a given combination of test factors cannot be attributed to random noise. In other words, if group b is a combination of test factors that give a statistically significant response to a variable, then $\sigma_{b}^{2}\gg \sigma^{2}$ [33]. The objective then is to compare $\sigma^{2}$ estimates by calculating variances within and between test factor groups [33].

### 3.2. Experimental Setup

For this experiment, a series of five independently manufactured PT samples were provided by Nu-tech Precision Metals Inc. The characteristics and measured dimensions of each of these samples are outlined in Table 1. As shown in Table 1, three different PT samples were obtained from the front ends of extruded and cold-worked PTs, while two were provided from two different PT back ends. To heat treat these PT samples, a Paragon PKM9 series industrial kiln was used. This device was capable of heating the PT samples up to the necessary 400 °C temperature, but could not support the steam pressure necessary to simulate the autoclave heat treatment conditions. The only gases that could be supported were atmospheric pressures of air and nitrogen. Since typical oxide layer thicknesses are on the order of 20 µm, as indicated by Price [27], this limitation was deemed acceptable, as it would introduce negligible lift-off variation effects into the voltage response of the EC probe. HTs on each of the PT samples were applied for 24, 48, and 72 h to give a total HT time of 144 h.

A total of six EC frequencies were selected for characterizing the significance of variations in EC frequency on measured PT resistivity. As per American Society of Testing Material (ASTM) E1004 [34], the standard approach for using ECT to measure a material’s electrical resistivity is to employ EC frequencies that give triple skin depths of penetration that are less than the material’s WT. A single skin depth, δ(ν), as a function of EC frequency, ν, is defined as the depth from an electrically conductive material’s surface at which the EC density decreases by a factor of $e^{-1}$ and is given by the following equation [35]:

(7) $$\delta \left(\nu \right)=\sqrt{\frac{\rho }{\pi \nu \mu }},$$

where ρ and μ are the material’s electrical resistivity and magnetic permeability, respectively. For Zr2.5%Nb materials, which are non-ferromagnetic, the magnetic permeability is equal to that of the free space given by the $\mu_{0}$ constant. Choosing EC frequencies that satisfy the triple skin depth (TSD) operating condition results in at least 95% of the surface EC density being attenuated through the thickness of the sample, thereby minimizing sensitivity to WT variations. Therefore, these six frequencies were chosen to satisfy this requirement, while still providing a range of skin depths through the PT WT. A comparison of the corresponding TSD values given by Equation (7) between each of the selected frequencies is shown in Table 2 below.

Additionally, since the interpretation of signals from ECT is based on relative comparisons, a calibration step is required to measure the voltage response of the probe on materials with similar resistivities that span the range above and below the unknown material’s resistivity [36]. Therefore, the characteristics of the PT samples that were chosen for this calibration step are listed in Table 3 below with each PT sample’s electrical resistivity being measured using the 4-point method, which only provides an average resistivity in the circumferential direction [14,17]. The thermal resistivity coefficients for the PT samples in this table were measured by Klein [14] to be $3.955\times 10^{-3}\;{{}^{\circ}C}^{-1}$.

**Figure 2:**
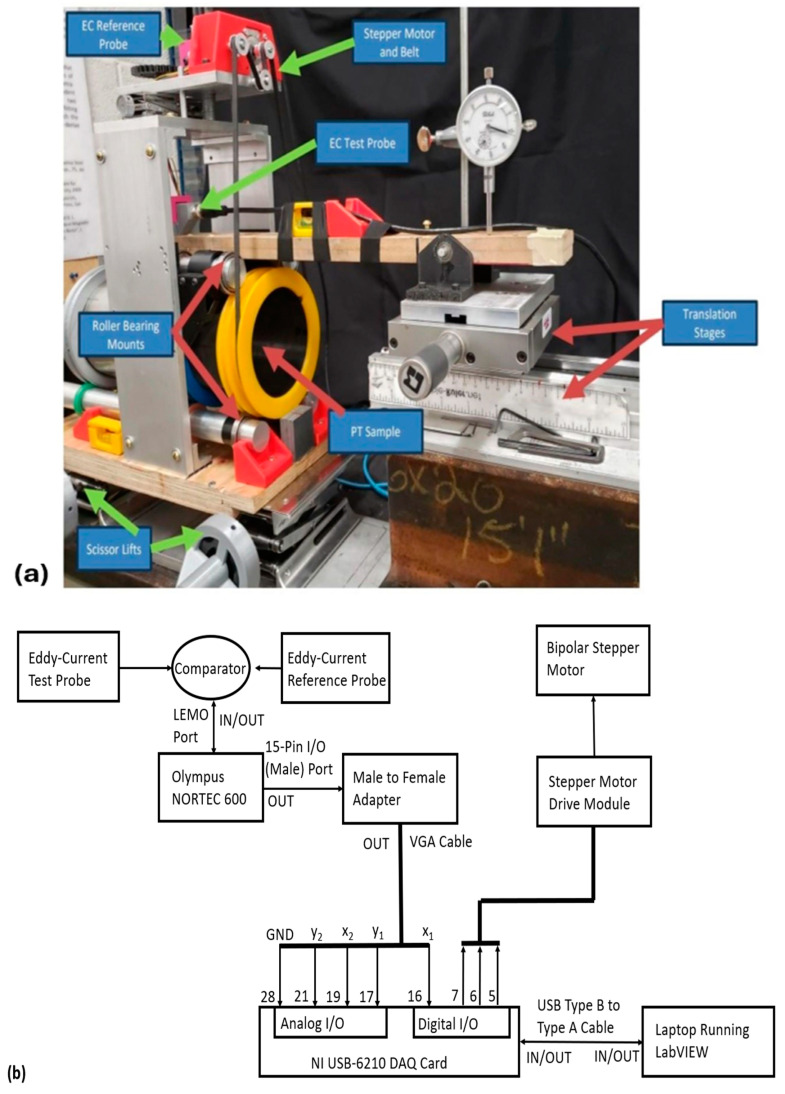
(**a**) Apparatus used for measuring electrical resistivity by circumferentially rotating a PT sample beneath a fixed absolute EC probe with annotations of each of the various components; and (**b**) a schematic block diagram of the electrical setup for operating the system and acquiring voltage response data from the EC test probe. The whole system is operated via a laptop running LabVIEW, with the data stored in a Microsoft Excel file.

A custom-built apparatus shown in Figure 2a was used for gathering circumferential scan data of the EC probe’s voltage response to circumferential variations in electrical resistivity in each of the PT samples listed in Table 1. This apparatus was designed to mitigate the effects of probe lift-off and tilt on its voltage response during each scan by ensuring that the probe was always held at a fixed position and maintained perpendicular contact with either the inner or outer PT surfaces. Circumferential rotation of the PT sample relative to the EC probe was automated using a belt-and-pulley system driven by an Adafruit bipolar stepper motor with the probe’s circumferential position encoded using a LabVIEW program to count the number of discrete shaft rotations of the stepper motor.

The electrical setup for operating and acquiring data from this apparatus is shown in Figure 2b. The effects of null-point temporal voltage drift were mitigated by using two EC probes with similar coil specifications in a differential configuration with the reference probe held in fixed contact over a titanium bar sample with a reported resistivity of 55.6 μΩ·cm [37]. The specifications of the coils in each of these probes are given in Table 4. The resistance and inductance values in this table were measured using a multimeter with uncertainty given by the formula [38]:

(8) δR=0.3%·R+0.02,

and an LCR meter, respectively. The uncertainty on the measured inductances was estimated using half its range of variation with frequency on the LCR meter. A NORTEC 600D EC Flaw Detector Instrument was used to energize the EC probes and acquire voltage response data from the differential combination of the EC probes with these data along the encoded circumferential position of the probe. This was then passed to a National Instruments Data Acquisition Card and laptop running LabVIEW to be saved in an Excel file.

**Figure 3:**
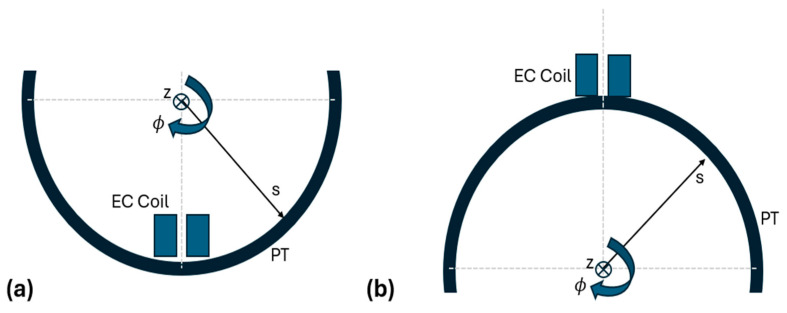
Schematic diagram (not to scale) illustrating the positioning of the EC coil over (**a**) the inner PT surface and (**b**) the outer PT surface in the apparatus of Figure 2a. Each of the axis directions are also shown labeled with s, ϕ, and z, corresponding to the radial, circumferential, and axial directions, respectively. The dashed lines in this figure are used to show the coil’s axis being perpendicular to the PT surface, a crucial design parameter of the apparatus for minimizing LO variational effects.

Figure 3 below provides a schematic diagram to illustrate the orientation of the EC probe in the apparatus of Figure 2a, relative to each of the PT surfaces. Figure 3a shows the probe configuration for conducting measurements from the inner surface, while Figure 3b shows the corresponding configuration for outer surface measurements.

Using this apparatus, circumferential voltage measurements were recorded at increments of 7.2° for a total of 50 measurements per revolution of the PT sample beneath the probe, which could be gathered in under a minute, while also providing sufficient resolution of any circumferential resistivity variations with each resistivity measurement corresponding to an average of the EC interactions within the microstructure beneath a projection of the probe’s cross-sectional area on the corresponding PT surface. Prior to performing any resistivity measurements using the EC probe with the apparatus, the phase angles of the input alternating current (AC) voltage signals for each frequency were adjusted so small lift-off effects of the test probe produced maximized changes in the negative direction of the horizontal (in-phase) voltage component. This setup is standard practice for conductivity testing using EC, as it results in changes in electrical resistivity producing a linear, positively correlated change in the response of the vertical voltage (quadrature) component of the nearby coil that is in contact with the surface [36]. In addition, the voltage component gain settings for each frequency were set to values to maximize the scale of the voltage variations for resistivity variations in the range of 51–57 μΩ·cm, while ensuring that they did not reach the saturation limit of ±5 V.

Measurement uncertainties for each PT surface and EC frequency from this apparatus were estimated using the steps outlined below, which assume an independent variance of probe voltage responses as a function of circumferential position around each of the PT samples.

1. Removing temporal voltage drift by assuming a linear variation with time between measurements at 0° and 360°.
2. Calculating the average value at each position across all trials and each of the corresponding differences of each data point from this overall positional average.
3. Calculating the mean variance of these calculated delta values from each positional average per degree of freedom (DOF) using the mean standard error (MSE) equation [33]:
  (9) $$\sigma_{\mu }^{2}=\frac{\sum_{i=1}^{N_{A}}\sum_{j=1}^{N_{B}}{\left(\Delta V_{y}^{j}\left(\theta_{i}\right)\right)}^{2}}{N_{A}\cdot {\left(N_{B}-1\right)}^{2}},$$
  where $N_{A}$ is the number of discrete circumferential positions, $N_{B}$ is the number of repeat measurements per position, and $DOF=N_{A}\cdot (N_{B}-1)$.
4. Determining the 95% confidence interval margins using the Student’s t-distribution [40], with the number of degrees of freedom as an input and scaling it by $\sigma_{u}$.

These reproducibility tests were performed by excluding PT Sample 5 from being heat-treated with the other PT samples so that repeat circumferential scan measurements of electrical resistivity could be compared, after each HT stage, between it and the other PT samples. This experimental setup with PT Sample 5 as a control parameter ensured that the results obtained on the other PT samples were as repeatable as possible with the apparatus used, since any intermittent effects on the resistivity measurements would, theoretically, present as significant discrepancies on measurements of the control tube.

In addition to taking measurements of PT electrical resistivity using ECT, the 4-point method [17] was also employed as an independent technique to verify the accuracy of the electrical resistivity measurements obtained from the ECT apparatus. For measuring the resistivity of PT samples, this method involved cutting a thin ring sample and then removing an additional notch from the circumference of the ring to ensure that the injected current could only flow in a single direction. Since the accuracy of the results obtained from the 4-point method is sensitive to the presence of zirconium oxide at the surfaces, which act as insulating boundary layers, only ring samples cut from each of the PT samples listed in Table 1, prior to commencing HT, were measured. A Keithley 6220 DC and AC Current Source [41] and Keithley 2182A Nanovoltmeter [42] were used for these measurements. The probes of the current source and voltmeter were then set up with the probes of the voltmeter placed 329.7° ± 0.5° apart on each PT ring sample (as measured using a protractor) and the probes of the current source placed on either side of the ring sample where the notch was removed. Razor blades were used as the point of contact between the probes and each PT ring sample to allow for more precise positioning and accurate estimation of the current travel path lengths.

## 4. Results

**Figure 4:**
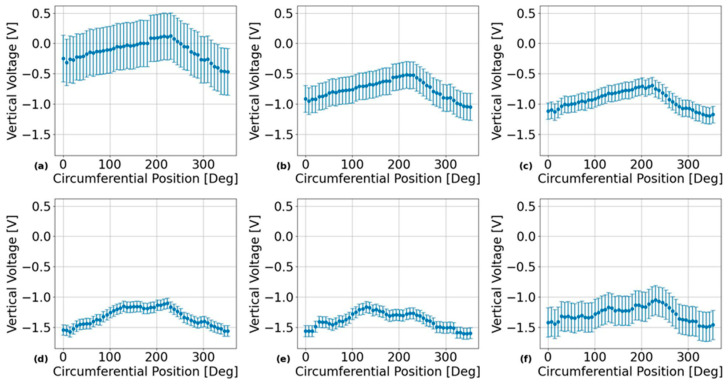
Results of applying the analysis procedure for quantifying experimental uncertainties in terms of 95% confidence bounds in the EC probe’s raw vertical voltage responses across multiple independent scans around the outer surface of PT Sample 5. These results show that, for most frequencies, the relative positional uncertainty is insignificant compared to the scale of the overall circumferential trends. The different subplots correspond to different inspection frequencies with: (**a**) 74 kHz, (**b**) 120 kHz, (**c**) 200 kHz, (**d**) 460 kHz, (**e**) 900 kHz, and (**f**) 1500 kHz.

**Figure 5:**
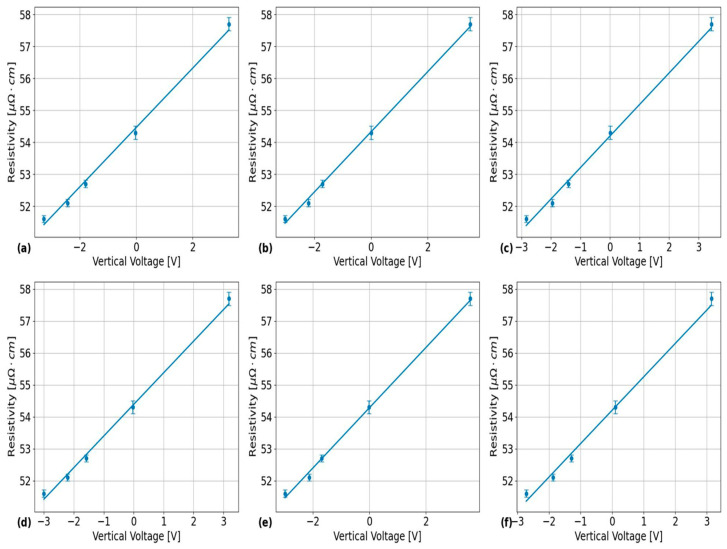
Sample plots showing the results from the calibration tests for converting the EC probe’s vertical voltage into outer PT surface electrical resistivity using the PT samples listed in Table 3. The different subplots in this figure correspond to the results obtained using EC frequencies of: (**a**) 74 kHz, (**b**) 200 kHz, (**c**) 900 kHz, (**d**) 120 kHz, (**e**) 460 kHz, and (**f**) 1500 kHz.

Sample results obtained by applying the analysis procedure indicated at the end of Section 3.2 (above) are shown in Figure 4. The results in this figure were obtained from the probe positioned over the outer surface of PT Sample 5 after being heat-treated for 24 h using the EC frequencies listed in Table 2 with the results in each subplot obtained from the EC frequencies of: (a) 74 kHz, (b) 120 kHz, (c) 200 kHz, (d) 460 kHz, (e) 900 kHz, and (f) 1500 kHz. Sample results from the calibration tests for each of the EC frequencies and PT sample surfaces are shown in Figure 5 for the outer surface placement of the EC probe over the calibration PT samples listed in Table 3. The corresponding linear regression analysis results from the sample plots in this figure are shown in Table 5. These calibration results were used to convert the probe’s vertical voltage response into an absolute resistivity using the following equation:

(10) $$\rho_{meas}\left(\phi ,\nu \right)=m_{RV}\left(\nu \right)\cdot V_{y}\left(\phi ,\nu \right)+b_{RV}\left(\nu \right),$$

for the corresponding inner or outer surface from which measurements were collected. In this equation, ϕ corresponds to the circumferential position, $V_{y}$ is the vertical voltage response, $m_{RV}$ is the resistivity-voltage (RV) calibration slope, and $b_{RV}$ is the y-intercept. The uncertainty, $\delta \rho_{meas}$, on the measured resistivity, $\rho_{meas}(\phi ,\nu )$, as a function of circumferential position, ϕ, and EC frequency, ν, was estimated using the derivative error propagation method in the QExPy module in Python with the uncertainties on each of the parameters as inputs [43].

A sensitivity analysis of the percentage contribution of each parameter’s uncertainty to the uncertainty of $\rho_{meas}(\phi ,\nu )$ was also conducted using the QExPy module. From this analysis, it was found that the contributions of all three parameters were found to be evenly distributed with average uncertainty contributions and 2σ standard deviations of: 60±40% for $\delta b_{RV}(\nu )$, 20±40% for $\delta m_{RV}(\nu )$, and 20±60% for ${\delta V}_{y}(\phi ,\nu )$.

The resistivities obtained from each data set were then averaged over all circumferential positions with the results being analyzed using a multi-parameter analysis of variance (MANOVA) methodology [33], with the test factors outlined at the start of Section 3.1. For a one-way analysis of variance (ANOVA), the relevant equations for this type of analysis are given by [33]:

(11) $${\bar{\rho }}_{j}=\frac{1}{n_{j}}\sum_{i}\rho_{ij},$$

(12) $$\bar{\rho }=\frac{1}{N}\sum_{i}\sum_{j}\rho_{ij},$$

(13) $$s_{W}^{2}=\frac{1}{m}\sum_{j}\frac{\sum_{i}{\left(\rho_{ij}-{\bar{\rho }}_{j}\right)}^{2}}{n_{j}-1},$$

(14) $$s_{A}^{2}=\frac{\sum_{j}n_{j}{\left({\bar{\rho }}_{j}-\bar{\rho }\right)}^{2}}{m-1},$$

where ${\bar{\rho }}_{j}$ is the sample mean for test factor combination group j containing $n_{j}$ resistivity values, $\rho_{ij}$ is the $i^{th}$ resistivity for test factor combination j, and $\bar{\rho }$ is the overall average in resistivity across all N measurements. The variances of the measured values for each test factor are then given by Equation (13), while the variances between test factors are given by Equation (14), where m defines the number of test factors. Extending these equations for MANOVAs is simply a matter of adding additional equations to calculate the variances within and between each of the test factor combinations.

For this analysis, the *statsmodels* module in Python [44] was utilized for performing the MANOVAs on the full-factorial data. In performing a MANOVA, multiple repeat measurements are required for all test factor combinations, so that the true variance in the response data can be determined. Repeat measurements were gathered on a single PT sample at one of its applied HT states as being representative of the variations for the remaining PT samples and applied HT states. As a result, the true variances for the resistivity measurements for the other PT samples and HT states could not be determined. This issue was resolved by using the average circumferential electrical resistivities from each of the test factor combinations for the multiple PT samples as the repeats. As a result, each grouping of this response variable in the full-factorial data had four repeat measurements from each of the PT samples listed in Table 1. PT Sample 5 was left out of this analysis, due to it having an incomplete data set for each of the applied HT levels, since this PT sample was being used as a control parameter for examining reproducibility.

**Figure 6:**
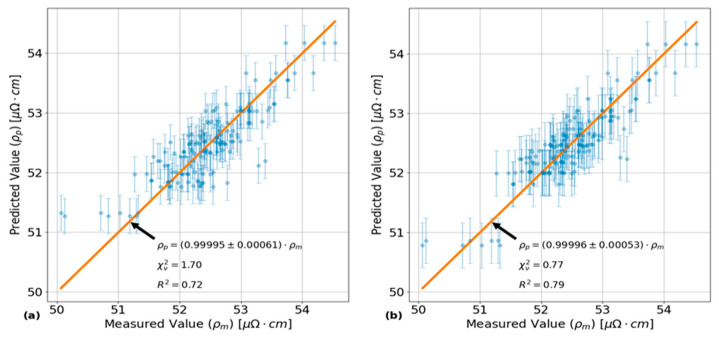
Plots showing the predictive capabilities of two different MANOVA models as a function of the measured average circumferential resistivity values. Each set of error bars corresponds to the 95% confidence bounds on the corresponding predicted value for a given measured value. The annotations show the parameters of the fitted lines that minimize the residual sum of squares between it and the corresponding data points, while being constrained to pass through the origin. Subplot (**a**) shows the amount of variance captured using a MANOVA model with the four most significant test factors and combinations, while subplot (**b**) shows the amount of variance captured when considering all test factors and combinations.

**Figure 7:**
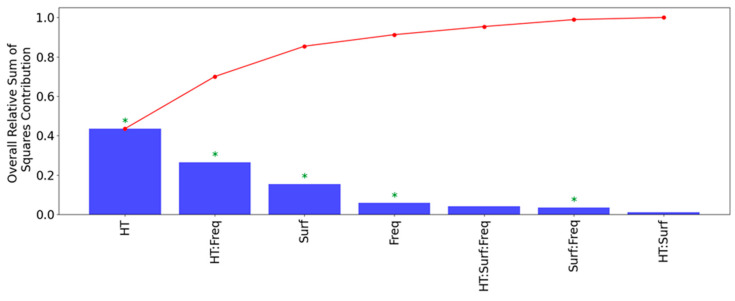
Pareto plot showing a ranking of the amount of relative variance captured by all the test factors and combinations included in the MANOVA model shown in Figure 6b. The plot in red shows the cumulative increase caused by each bar going from left to right. The ‘*’ symbols indicate the parameters that are statistically significant relative to the null hypothesis with F-test values less than 0.05.

The results of this MANOVA analysis are shown in Figure 6 and Figure 7. The plot in Figure 6a shows the results of the predictive capabilities of a MANOVA model using only the four highest test factors and combinations shown in the Pareto plot of Figure 6. In the Pareto plot of Figure 7, groups of test factors are denoted by factor names separated by a “:”, with “Freq” and “Surf” being shorthand for the eddy current frequency and probe surface placement, respectively. The plot in Figure 6b shows the predictive capabilities of a separate MANOVA model using all the test factors and combinations shown in Figure 7.

**Figure 8:**
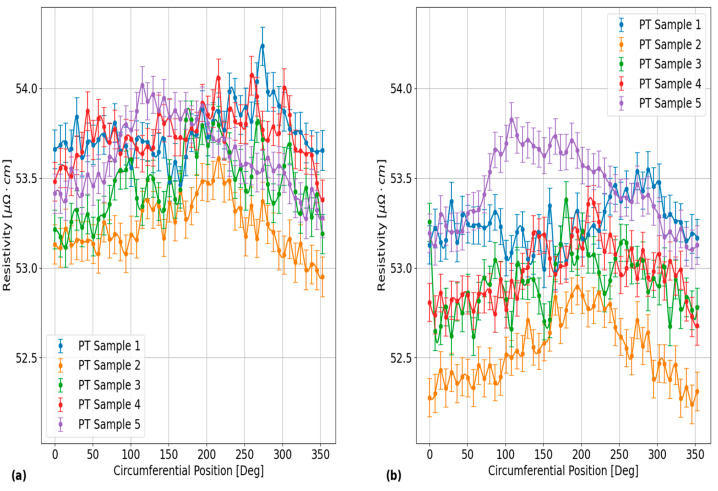
Plots showing a comparison of the EC probe’s vertical voltage response to electrical resistivity variations around the outer surface circumference in each of the PT samples after 24 h of applied HT at 400 °C. The different subplots correspond to the results obtained using the EC frequencies of: (**a**) 120 kHz and (**b**) 900 kHz. As shown, the circumferential variations differ between each of the PT samples, with similar trends between different EC frequencies. These results are representative of the results obtained for the circumferential variation from the inner surface circumferences of each of these PT samples.

The plots in Figure 8 provide an illustration of the differences in circumferential resistivity trends between each of the PT samples in this experiment after being heat-treated for 24 h at 400 °C. These results were obtained with the EC probe positioned over the outer surface of each of these samples using EC frequencies of: (a) 120 kHz and (b) 900 kHz. Each data point in these plots corresponds to an average of all repeat measurements at the given circumferential position, with the error bars corresponding to the error propagated results in Equation (10) of the 95% confidence bounds, as determined using the data analysis steps on the reproducibility results from PT Sample 5. As shown, the trends in circumferential resistivity are fairly consistent between the EC frequencies for each PT sample, but the results do vary between each of the samples. These results are representative of the results obtained from the inner surfaces of the PT samples. The maximum 2σ standard deviation of all the circumferential resistivity measurements between each of the PT samples in this figure is 0.39 μΩ·cm and 0.36 μΩ·cm, for EC frequencies of 120 kHz and 900 kHz, respectively.

**Figure 9:**
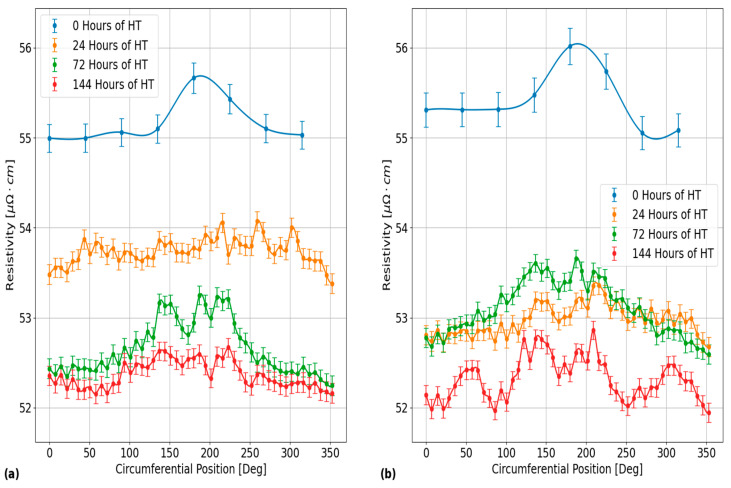
Plots showing a comparison of the EC probe’s vertical voltage response to electrical resistivity variations around the outer surface circumference of PT Sample 4 after each applied HT stage at 400 °C. The different subplots correspond to the results obtained using the EC frequencies of: (**a**) 120 kHz and (**b**) 900 kHz. As shown, the circumferential variations differ between each of the HT stages and there is a general decrease in the average circumferential resistivity between HT stages that is most apparent for the results obtained using an EC frequency of 120 kHz. These results are representative of the results obtained for the circumferential variation from the inner surface of this PT sample, as well as the results of all the other PT samples.

Furthermore, the plots in Figure 9 provide an illustration of the effects of HT on the circumferential resistivities in each of the PT samples. The results in this figure were obtained for the EC probe located on the outer surface of PT Sample 4. The inner surface results are not shown in this plot, but they exhibit similar degrees of circumferential variation to the outer surface results. The different subplots in Figure 9 correspond to the results that were obtained using EC frequencies of: (a) 120 kHz and (b) 900 kHz. The data points and error bars for the plots in Figure 9 were determined using the same procedure as outlined for the plots in Figure 8. From this figure, the maximum 2σ standard deviation of all circumferential resistivity measurements between each of the HT stages is 0.61 μΩ·cm and 0.66 μΩ·cm, for EC frequencies of 120 kHz and 900 kHz, respectively. Another result that can be seen from the plots in Figure 9 is that the circumferential variation in resistivity in PT Sample 4 was similar between each of the applied HT stages.

**Figure 10:**
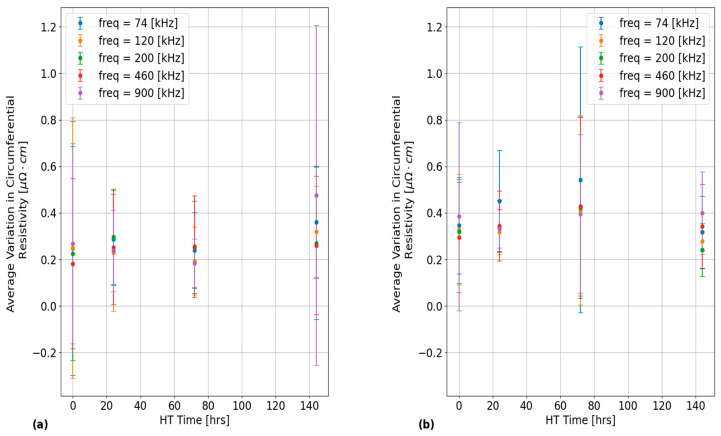
Plots of the average 2σ standard deviation in circumferential resistivity between PT samples that were obtained using various EC frequencies for: (**a**) the inner PT surface placement; and (**b**) the outer PT surface placement of the EC probe. The error bars correspond to the 2σ standard deviation of each of these values across all PT samples.

In addition, the plots in Figure 10 provide quantifications of the 2σ standard deviation of each set of circumferential resistivity measurements with these results being averaged across all PT samples for each EC frequency and probe surface placement. The error bars in each of these plots correspond to the 2σ standard deviation of 2σ circumferential variation values across all PT samples. The error bars in Figure 10 quantify the amount of statistical variation within each of the PT samples and do not represent the experimental uncertainty. The results in subplot (a) were obtained with the probe placed over the inner PT surface, while the subplot (b) results were obtained with the outer PT surface placement. As shown, similar results were obtained between each of the EC frequencies and probe surface placements on the PT samples. There is also a weak correlation between variations in circumferential resistivity and applied HT time at 400 °C. The large error bars in these plots indicate that the size of the circumferential resistivity variations in each of the PT samples are mainly dictated by inherent differences between each of these samples, which could be a result of variability in manufacturing history.

**Figure 11:**
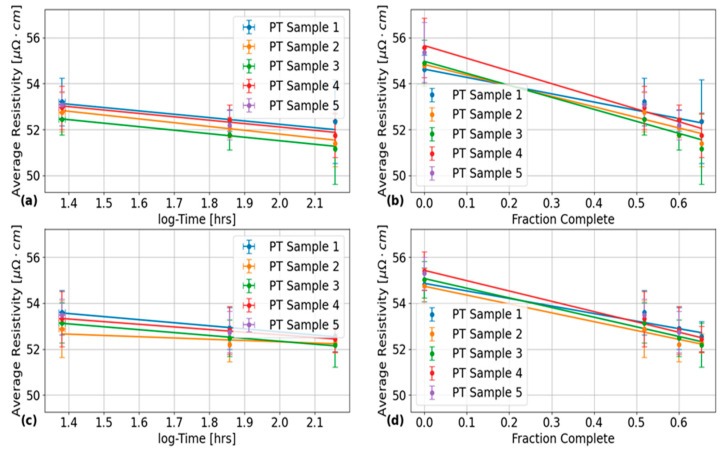
Plots of the average electrical resistivity measured after 24, 72, and 144 h of total applied HT at 400 °C on various PT samples as a function of (**a**,**c**) log time and (**b**,**d**) fraction complete. Each data point represents an average of the EC response data gathered at various circumferential positions and frequencies. The results in (**a**,**b**) were obtained for the inner surface data, while the results in (**c**,**d**) were obtained for the outer surface data. The error bars correspond to the results obtained for a 2σ standard deviation across all measurements. The added trendlines were fit to minimize the sum of the residual square error between it and its corresponding PT sample dataset.

The final set of results that were generated from this analysis are shown in Figure 11, which plots the average electrical resistivity across all circumferential positions and EC frequencies for: (a), (b) the inner PT surface; and (b), (d) the outer PT surface as a function of: (a), (c) HT time in log hours; and (b), (d) fraction complete. This fraction complete scale corresponds to the results obtained using the Avrami fit parameters obtained at the end of Section 2 in Equation (1) with Y(t) now corresponding to the fraction complete variable by the previously discussed assumptions. The vertical error bars in these plots correspond to the 2σ error estimates using the standard deviation formula [33]. The horizontal error bars in Figure 11a,c correspond to the results of propagating the estimated uncertainty in an HT time of 30 min, which corresponds to the amount of time necessary for the kiln to heat up to 400 °C. In Figure 11c,d, no horizontal error bars are shown due to division by zero computation errors being encountered in attempting to propagate the uncertainties in the Avrami fit parameters in Equation (1). Line of best fits are shown in each of the subplots in Figure 11, which were determined using linear regression analysis with the quantitative results given in Table 6 and Table 7, for the fits in Figure 11a,c, and Figure 11b,d, respectively. The plots in Figure 11a,c and Figure 11b,d show two different ways of presenting the same results. The difference in number of data points between each set of plots arises because the data points at zero HT time (zero fraction complete) are undefined on a logarithmic time scale.

A comparison of the results that were obtained from the two 4-point method measurement techniques on the non-heat-treated ring samples from each of the PT samples in Table 1 and the results that were obtained from the ECT method are shown in Table 8 below. The derivative error propagation method in the QExPy module [43] in Python was used to calculate the uncertainties on each of the 4-point method results.

The results in Table 8 for the ECT method were obtained by averaging all the resistivity measurements made with this method for each PT sample with the uncertainty calculated as the 2σ standard deviation of the measurements on each PT sample. Therefore, these results represent an average of all resistivity measurements through the WT of each PT sample with the uncertainties quantifying the amount of variation within a 95% confidence bound of each average.

As shown in Table 8, the measured resistivities using ECT agree within error of the resistivities measured using the 4-point method. Therefore, it can be concluded that the as-built apparatus in Figure 2 does indeed give accurate measurements of electrical resistivity in PTs. The results from both methods also show little to no variation, beyond the uncertainty confidence bounds between PT samples, which is an indicator of consistency in the as-manufactured PT sample properties for this set of PTs. The results are also consistent with those reported by Bennett et al. [28] for a different PT sample.

## 5. Discussion

The results of the Pareto plot in Figure 7 show that the set of test factors and combinations that capture the majority of the variance in the full-factorial average circumferential resistivity data are:

1. Heat treatment (HT);
2. HT and EC frequency;
3. Probe inner or outer surface placement;
4. EC frequency.

The effects of each of these test factor combinations on the average circumferential resistivity in PTs are also all statistically significant, as the F-test results for the comparison of variances between sample groups are all orders of magnitude less than the commonly used critical value of 0.05 [33]. Therefore, the null hypothesis can be rejected for each of these listed test factor groups as there is a vanishingly low probability that these effects are the result of random influences.

These four test factor groups account for 72% of the total variance in the data, which is shown by the annotated correlation coefficient in Figure 6a. If all test factors and combinations are considered as shown in Figure 6b, the maximum amount of the total variance that can be captured is 79%. As a result, there is about 21% of unexplained variance in the resistivity data that cannot be attributed to any of the test factors or combinations. This 21% of variance is likely attributable to inherent differences in microstructure between each of the PT samples due to variations in the manufacturing conditions. One such parameter that was not accounted for as a test factor in this analysis was the effect of having PT samples cut from the different extrusion ends of the manufacturing process. This extrusion process is expected to introduce variations in the average grain texture between the front and back end samples listed in Table 1, as indicated by Holt [3]. The significance of the HT and EC frequency test factors on measurements of average circumferential electrical resistivity in PTs using ECT agree with the expectations outlined in the introduction as well as the through-thickness resistivity measurements obtained by Thorpe et al. [23].

However, an anomalous result is the apparent significance of the combination of HT and EC frequency on the circumferential electrical resistivity in PTs as the interaction of these two test factors accounts for roughly 24% of all the variation in the data. Since different EC frequencies result in different penetration depths into a conductive material, as per the skin effect [36], this result from the MANOVA indicates that the HT methodology used in this experiment generated variations in the radial resistivity trends in each of the tested PT samples, as shown in Figure 7. Further evidence of this conclusion is shown in Figure 8, with the different subplots showing that the HT process did not result in uniform rates of decrease in resistivity between the EC frequencies of 120 kHz and 900 kHz. This finding is unexpected, since the industrial kiln used for applying HTs should have been able to reach a uniform temperature everywhere within the HT compartment. It is possible that these radial resistivity variations in the PT samples are a result of the initial temperature gradient that forms between the PT surfaces and the midpoint in its WT. However, further investigation on the conditions immediately after the hot-extrusion process is needed to model the rate of heat transfer in PTs. Another limitation of this study is the lack of characterization of the microstructure of these PT samples before and after each stage of HT. As a result, the exact microstructural causes of these radial resistivity variations are unknown.

An unexpected result from the quantification of experimental uncertainties in the reproducibility plots shown in Figure 4 is that the size of the error bars seems to randomly vary with the EC frequency, with the error bars being largest at the lowest frequency of 74 kHz. The only source of variation from this experiment that could explain this result is the lack of consistency in the resistivity-voltage (RV) calibration slope values as a function of EC frequency, as shown in Table 5. As such, further investigations are required here to examine whether the reproducibility of circumferential resistivity scans in PTs can be made more consistent between EC frequencies using a consistent RV sensitivity for all frequencies. Another notable result from Figure 7 is the apparent significance of the PT surface placement on the average circumferential resistivity measured by the EC probe. However, no statistically significant differences in average circumferential resistivity were observed between the different surface placements of the EC probe over each of the PT samples. Therefore, its likely that the significance of this parameter comes from Equation (6) under the null hypothesis not being satisfied for the variances in average circumferential resistivity between each of the PT surfaces. However, since in-situ microscopy examinations of the microstructure were not performed on each of these PT samples between HTs, it is unknown whether these effects have microstructural correlations with inner and outer surface differences in $\beta_{Zr}$ ribbon thickness, as reported by Thorpe et al. [23]. If these results are indeed an effect of the microstructure, then they would indicate that calibrations of EC probe resistivity measurements can be significantly improved by using PT samples with known resistivities, as measured from the outer and inner surfaces. To obtain such measurements, one would need a set of unalloyed and isotropic samples with the same geometric shape as the PTs to compare resistivities between the inner and outer surfaces in a given PT test sample.

From the plots in Figure 11, the maximum 2σ standard deviations in electrical resistivity measurements in the circumferential direction of each of the PT samples is about ±1.2 μΩ·cm from the inner PT surface. Therefore, based on the sensitivity analysis of the concentric tube model by Klein et al. [14], it can be inferred that circumferential variations in the resistivity of newly installed PT samples in the fuel channels of CANDU^®^ reactors introduce PT-CT gap errors of up to 0.12 mm and 0.18 mm for EC inspection data obtained using frequencies of 4 and 8 kHz from the inner PT surface, respectively.

The final set of results in Figure 11a and Table 6 show that HT at 400 °C consistently results in an average decrease in resistivity across all PT samples of about $1.53\pm 0.08\;\frac{\mu \Omega \cdot cm}{\log\left(hr\right)}$ and $1.1\pm 0.4\frac{\mu \Omega \cdot cm}{\log\left(hr\right)}$ from the inner and outer PT surfaces, respectively. The uncertainties on these values correspond to the 2σ standard deviation of each of the results. The outer surface results agree within error of the value reported by Bennett et al. [28], but the inner surface results do not, which could be due differences in the $\beta_{Zr}$ ribbon thickness near each of the surfaces as found from the metallographic analysis of the microstructure in the axial-transverse cross-section of a non-heat-treated PT sample by Thorpe et al. [23]. However, the statistical significance of the results in Figure 11 are low due to the 2σ error bars in these plots showing significant overlap with each other at each of the plotted data points. The ranking of test parameter influences on average circumferential resistivity in PTs in Figure 7 also shows that the interaction term between the HT and PT surface has the least significant effect on the variance in these values.

Another result from Figure 11b is that the decomposition of the $\beta_{Zr}$ phase in Zr2.5%Nb PTs results in a change in resistivity of about $-4\pm 1\frac{\mu \Omega \cdot \mathrm{cm}}{\Delta f_{c}}$, where $\Delta f_{c}$ is the change in fraction complete. The y-intercepts from these fitted lines are also generally in good agreement with those extrapolated from the fitted lines in the plots of Figure 11a,c, which indicates that for each PT sample and probe surface placement, there is a strong correlation with the linear trend for all data points. The consistency of these results between each of the PT surfaces indicates that the phase transformation of $\beta_{Zr}$ to $\beta_{Nb}$ occurs at roughly equal rates for the two surfaces.

The results presented here demonstrate the utility of using ECT to perform high-resolution (radial and circumferential) resistivity measurements, even under conditions of growth of an insulating material oxide layer, relative to what would be possible by 4-point resistivity measurements. Future work would be to develop the inversion of multi-frequency EC measurements to extract depth dependence of resistivity, but this would require non-alloyed and isotropic calibration standards.

## 6. Conclusions

A full-factorial experiment was used to examine the statistical significance of EC test parameters and manufacturing conditions on the electrical resistivity of Zr2.5%Nb PTs. The parameters that accounted for the majority of variance captured in the data were: (1) HT; (2) HT and EC frequency; (3) probe inner or outer surface placement; and (4) EC frequency. About 21% of the unexplained variance was likely a result of inherent differences in microstructure caused by variations in manufacturing process conditions between the PT samples. The significance of the PT surface parameter was attributed to differences between PT surfaces in variance of the average circumferential resistivity. Measurements of resistivity in the circumferential direction showed variation of up to ±2.3% of a PT’s average resistivity for either surface, which has implications for PT-CT gap measurement accuracy that currently assumes a constant resistivity [15]. Multi-frequency ECT in the circumferential direction across multiple PT samples showed that HT causes the average PT resistivity to decrease at a rate of $1.53\pm 0.08\;\frac{\mu \Omega \cdot cm}{\log\left(hr\right)}$ and $1.1\pm 0.4\frac{\mu \Omega \cdot cm}{\log\left(hr\right)}$ for the inner and outer PT surfaces, respectively. These results are correlated with reported differences in average $\beta_{Zr}$ ribbon thickness in the axial-transverse cross-section in Ref. [23], and therefore provide evidence of a radial resistivity variation being created due to HT. It was also found that about 24% of the variance could be explained by the interaction between the test factors of HT and eddy current frequency, which is further evidence of the HT process impacting radial resistivity variations in the PT samples. These results provide a more nuanced understanding of the changes in electrical resistivity of Zr2.5%Nb PTs caused by HT time at 400 °C and have relevance for characterizing electrical resistivity in PTs used to calibrate the EC-based PT-CT gap probe response.

## Figures and Tables

**Table 1:** PT test sample specifications, with size measurements made using a digital pipe micrometer for WT, and digital caliper for OD. Error values on WT measurements correspond to the standard deviation of the various circumferential measurements. The uncertainty on the OD values is estimated to be ±0.03 mm based on the caliper’s datasheet.

PT Sample Number	Side of Tube from Manufacturing Process	OD (mm)	Average Front Side WT (mm)	Average Back Side WT (mm)
1	Front	112.20	4.44 ± 0.02	4.39 ± 0.01
2	Front	112.74	4.49 ± 0.08	4.52 ± 0.08
3	Front	112.10	4.56 ± 0.06	4.5 ± 0.1
4	Back	112.20	4.5 ± 0.1	4.5 ± 0.1
5	Back	112.43	4.65 ± 0.06	4.6 ± 0.1

**Table 2:** List of selected EC inspection frequencies and corresponding TSD values calculated using the skin depth equation [35]. These frequencies were used for experimentally investigating the presence of any radial gradients in electrical resistivity in the PT samples provided by Nu-tech Precision Metals Inc.

Frequency (kHz)	Triple Skin Depth (mm)
74	4.00
120	3.14
200	2.43
460	1.60
900	1.15
1500	0.89

**Table 3:** Calibration Sample (CS) PT specifications, with resistivities being measured using the 4-point method and size measurements made using a 150 mm Mastercraft digital caliper with an accuracy of ±0.03 mm.

PT Calibration Sample No.	ρ (20 °C) (μΩ·cm)	OD (mm)	ID (mm)
CS1	51.1 ± 0.1	112.12	104.05
CS2	51.6 ± 0.1	112.74	103.70
CS3	52.2 ± 0.1	112.10	104.25
CS4	53.8 ± 0.2	112.20	104.16
CS5	57.2 ± 0.2	112.20	104.15

**Table 4:** Characteristics of the as-built EC coils for use in the measurement and reference eddy current probes. The source for the relative permeability value of the ferrite cores is [39].

Parameter	Measurement Probe Value	Reference Probe Value
Wire Gauge (AWG)	36	36
ID (mm)	2	2
OD (mm)	5	5
Length (mm)	3	3
Number of Turns	80	80
Relative Permeability of Ferrite Core	25	25
Inductance (µH)	80 ± 5	67.5±2.5
Resistance (Ω)	1.80 ± 0.03	1.50±0.02
Capacitance (pF)	≤20	≤20

**Table 5:** Sample calibration test results showing the slope (Resistivity-Voltage (RV) slope) and intercept (RV intercept) values from a weighted linear regression fit to the EC probe’s vertical voltage response to each of the calibration tube samples. These results are separated by PT surface with ID for inner surface and OD for outer surface, and EC inspection frequency.

PT Surface (ID or OD)	Frequency (kHz)	RV Slope (µΩ·cm/V)	RV Intercept (µΩ·cm)
ID	74	0.92 ± 0.06	54.6 ± 0.2
	120	1.00 ± 0.07	54.5 ± 0.2
	200	0.99 ± 0.05	54.5 ± 0.1
	460	0.96 ± 0.05	54.4 ± 0.1
	900	1.06 ± 0.06	54.4 ± 0.1
	1500	1.14 ± 0.09	54.5 ± 0.2
OD	74	0.93 ± 0.04	54.5 ± 0.1
	120	0.99 ± 0.05	54.4 ± 0.1
	200	0.94 ± 0.04	54.33 ± 0.08
	460	0.95 ± 0.04	54.28 ± 0.09
	900	0.99 ± 0.05	54.2 ± 0.1
	1500	1.05 ± 0.07	54.2 ± 0.1

**Table 6:** Quantitative results for the line of best fits shown in Figure 11a,c, with $R^{2}$ corresponding to the correlation coefficient of each of the line fits.

Probe Surface Placement	PT Sample Number	$\mathrm{Slope}\;(\frac{\mu \Omega \cdot cm}{log\left(hr\right)})$	y-Intercept (μΩ·cm)	$R^{2}$
Inner Surface	1	−1.5 ± 0.5	55.1 ± 0.9	0.880
	2	−1.7 ± 0.3	55.1 ± 0.6	0.982
	3	−1.5 ± 0.2	54.6 ± 0.3	0.990
	4	−1.5 ± 0.4	55.1 ± 0.6	0.964
Outer Surface	1	−1.33 ± 0.08	55.4 ± 0.2	0.996
	2	−0.6 ± 0.5	53 ± 1	0.777
	3	−1.3 ± 0.1	54.9 ± 0.2	0.991
	4	−1.15 ± 0.07	54.9 ± 0.1	0.995

**Table 7:** Quantitative results for the line of best fits shown in Figure 11b,d, with $R^{2}$ corresponding to the correlation coefficient of each of the line fits.

Probe Surface Placement	PT Sample Number	$\mathrm{Slope}\;(\frac{\mu \Omega \cdot cm}{\Delta f_{c}})$	y-Intercept (μΩ·cm)	$R^{2}$
Inner Surface	1	−3.6 ± 0.4	54.6 ± 0.2	0.954
	2	−4.6 ± 0.6	54.8 ± 0.3	0.956
	3	−5.2 ± 0.5	55.0 ± 0.3	0.980
	4	−5.5 ± 0.6	55.7 ± 0.3	0.986
Outer Surface	1	−3.3 ± 0.5	54.9 ± 0.3	0.932
	2	−3.9 ± 0.3	54.7 ± 0.2	0.983
	3	−4.2 ± 0.4	55.1 ± 0.2	0.982
	4	−4.5 ± 0.2	55.4 ± 0.1	0.992

**Table 8:** A Comparison of the results of the electrical resistivity measurements that were obtained by using the 4-point method on non-heat-treated ring samples and ECT on the PT samples listed in Table 1. The uncertainties on each of the given values for the 4-point method results correspond to the 2σ confidence bounds that were obtained by using the derivative error propagation method in the QExPy module in Python. The resistivities given for the ECT method correspond to an average of all the resistivity measurements that were obtained on each PT sample with the uncertainties corresponding to the 2σ standard deviations.

PT Sample	4-Point Method Resistivity (μΩ·cm)	ECT Method Resistivity (μΩ·cm)
1	54.5 ± 0.2	54.7 ± 0.8
2	54.2 ± 0.2	54.8 ± 0.8
3	54.5 ± 0.2	55 ± 1
4	54.6 ± 0.2	56 ± 1
5	54.4 ± 0.2	55 ± 1

## Data Availability

The data are available as published in the tables and figures of this paper.
